# The value of interdisciplinary exposure: how university disciplinary diversity shapes research productivity of PhD students

**DOI:** 10.3389/fpsyg.2026.1797110

**Published:** 2026-06-17

**Authors:** Shijiao Cao

**Affiliations:** Business School, Chengdu University of Technology, Chengdu, China

**Keywords:** disciplinary diversity, interdisciplinary exposure, interdisciplinary research, PhD student, research productivity

## Abstract

**Purpose:**

PhD students at different universities face varying opportunities for interdisciplinary exposure, which may influence their academic performance. This study empirically investigates how university disciplinary diversity, which creates a context for the sharing and transfer of interdisciplinary knowledge, is associated with PhD student research productivity.

**Methods:**

This study proposes a novel measure of university-level disciplinary diversity and employs Poisson regression to examine its impact on PhD students’ research productivity, controlling for a range of individual-, supervisor-, and institution-level factors, based on a sample of 1,418 accounting dissertations submitted to Chinese universities between 1999 and 2022.

**Results:**

While a linear model points to an aggregate negative effect of university disciplinary diversity on PhD students’ research productivity, detailed analyses reveal a more nuanced inverted U-shaped relationship, suggesting that moderate levels of disciplinary diversity facilitate research productivity, and that the optimal level of diversity increases with a university’s overall resources. Further analyses uncover that the negative effect is concentrated in universities with greater disciplinary imbalance or those that expand into too many irrelevant disciplines. Additionally, evidence indicates that PhD students at universities with higher disciplinary diversity are more likely to engage in interdisciplinary research and produce work with greater impact.

**Discussion:**

This study sheds new light on how interdisciplinary exposure is associated with PhD students’ research productivity and offers practical implications for doctoral training. Engaging with interdisciplinary knowledge may help students identify innovative research questions and produce novel insights, underscoring the value of maintaining adequate disciplinary diversity at the university level.

## 1 Introduction

As undergraduate education has become massified worldwide in recent decades, doctoral education has followed to experience substantial growth (Pearson et al., 2008; Horta et al., 2018; Corsini et al., 2022). For example, between 1998 and 2008, the number of science doctorates awarded annually in OECD countries increased by nearly 40%, reaching approximately 34,000 (Cyranoski et al., 2011). In China, the number of doctoral entrants has markedly increased from 32,093 in 2001 to 153,275 in 2023, according to the statistics reports from the Ministry of Education of China. Against this backdrop, PhD students have been contributing to an increasingly large share of new knowledge production. However, the expansion of doctoral education has intensified peer competition, leading students to prioritize academic publications, as research productivity during the PhD is widely regarded as a key indicator of future academic success (García-Suaza et al., 2020; Lindahl et al., 2021; Li and Fernandez, 2025). While existing studies have explored the determinants of PhD students’ research productivity (Corsini et al., 2022; Roche, 2022; Lindahl, 2023; Li and Fernandez, 2025; Pan et al., 2025), far less attention has been paid to the university that provides access to academic resources and shapes their research outcomes.

Regarding scientific research, when it delves deeply into a specific field, new innovations become difficult to create, and breakthroughs sometimes emerge at the intersection of knowledge from different disciplines (Yu et al., 2024). At the same time, many real-world problems are not within-discipline problems, and solving them requires knowledge beyond the scope of a single discipline (National Academy of Sciences, National Academy of Engineering, Institute of Medicine, 2005; Ledford, 2015). Therefore, interdisciplinary research has long been emphasized by both the academic community and science policymakers (National Academy of Sciences, National Academy of Engineering, Institute of Medicine, 2005; Ledford, 2015; Wu et al., 2015; Yu et al., 2024). As the primary incubators of PhD students, universities are naturally well-suited to facilitate the sharing, transfer, and integration of knowledge across disciplines. Universities differ in their disciplinary diversity, and as they continuously adjust their discipline portfolios, PhD students experience varying levels of access to interdisciplinary exposure. While supervisors and home departments are more closely related to PhD students, they offer a much narrower scope of knowledge exposure. A supervisor’s research diversity, even when high, typically spans only a few adjacent subfields, and a department’s resources remain largely bounded by its home discipline. In contrast, a university’s disciplinary portfolio can encompass fundamentally distinct knowledge domains, such as political science, sociology, psychology, and computer science, which are often critical for generating novel research (Wu et al., 2015; Ledford, 2015). Moreover, university-level diversity is more exogenous than supervisor- or department-level diversity, as the latter are partly shaped by the former. Furthermore, understanding how university disciplinary diversity is associated with PhD student research outcomes would be useful to inform university strategic planning and doctoral education reform. Taken together, understanding the role of university-level disciplinary diversity is of vital significance for higher education research.

A university with diverse disciplines can affect its students in two ways. On the formal side, there are interdisciplinary courses and co-supervision arrangements with professors from other departments. On the informal side, students benefit from cross-departmental seminars, research centers, casual conversations with peers from different fields, library collections that reach beyond their home discipline. While disciplinary diversity is likely to foster knowledge sharing and integration, it may come at the cost of resource dilution, given that the total amount of resources of a university is limited. Considering that academic research is a knowledge- and resource-intensive activity, this leaves the question of whether and how disciplinary diversity affects the research productivity of PhD students an open one. Therefore, this study is set forth to explore whether the university-level disciplinary diversity promotes PhD student research productivity. Specifically, this study pursues three important research questions:

RQ1: How is university disciplinary diversity associated with PhD student research productivity?

RQ2: Do the balance and relevance of disciplinary diversity moderate the effect on student research productivity?

RQ3: How does university disciplinary diversity affect the likelihood of PhD students engaging in interdisciplinary research and their research impact?

To answer these questions, this study proposes a method to measure the university-level disciplinary diversity, which is based on the research areas in which a university publishes papers in a rolling four-year window that are indexed in the InCites database. Given the substantial differences in research styles and publication processes across disciplines (Pull et al., 2016; García-Suaza et al., 2020), this study narrows the research scope to a social science discipline, namely, accounting, for three reasons. First, accounting has long been one of the largest majors in Chinese higher education. This large scale makes accounting education an important research subject, and findings on accounting doctoral training can therefore benefit a substantial number of universities and students. Second, accounting research has a strong interdisciplinary tradition. Prior studies have documented that accounting scholars frequently draw on theories, methods, and data from economics, management, psychology, sociology, and even computer science (Hopwood, 1983; Xu et al., 2023). This makes accounting a suitable case for examining how university-level disciplinary diversity affects doctoral research. Third, focusing on a single discipline holds constant the substantial differences in publication norms, collaboration patterns, and citation practices that exist across fields, thereby enhancing internal validity. This study collects 1,418 doctoral dissertations in accounting submitted between 1999 and 2022 from CNKI (China National Knowledge Infrastructure) and manually extract useful information, including the number of refereed-journal papers published during their PhD studies, the time to degree, among others, from each of them. Poisson regressions that control for a wide range of confounding factors, including, for example, the gender of the student, the duration of the study, the rank and title of the supervisor, and the characteristics of the university, are used to empirically investigate the relationship between university disciplinary diversity and student research productivity.

This study makes two interrelated contributions to the literature on higher education and research productivity. First, the study advances the literature on doctoral education by identifying interdisciplinary exposure as a key determinant of PhD student research productivity. It demonstrates that, if adequate resources are available, exposure to interdisciplinary knowledge can enhance research output. This not only underscores the value of contextual resources beyond individual attributes but also reframes interdisciplinary exposure as a practical catalyst for academic performance. Second, it addresses a critical gap in our understanding of the substantive consequences of university disciplinary diversity. While universities are actively reconfiguring their disciplinary portfolios, existing research has largely focused on structural or symbolic aspects of diversity, with limited attention to its functional outcomes. By empirically examining how the university-level disciplinary diversity shapes student-level research productivity, this study moves beyond descriptive accounts to uncover the mechanisms through which disciplinary diversity translates into academic outcomes. The findings are thus highly relevant to program directors designing doctoral training models and to doctoral students navigating their research in increasingly complex academic landscapes.

## 2 Literature review and theoretical analysis

### 2.1 Previous literature on PhD student research productivity

PhD students are accounting for an increasingly large proportion of newly generated knowledge (Larivière, 2012; Lindahl, 2023), and the research outputs produced during their PhD studies are crucial for their competitiveness in job applications, grant proposals, and tenure evaluations, and are also found to be predictive of their future academic success (García-Suaza et al., 2020; Lindahl, 2023; Li and Fernandez, 2025; Sefika et al., 2025). Therefore, significant effort has been devoted to understanding the factors that influence PhD students’ research productivity. To provide a brief review of relevant literature, this study classifies the factors explored into three categories: individual-level factors, supervisor-related factors, and contextual and institutional factors.

At the individual level, gender is one of the most studied factors. Prior studies find that female scientists, including PhD students, have fewer publications compared to their male counterparts (Prpić, 2002; Lindahl et al., 2020), which is known as the “productivity puzzle.” Lubienski et al. (2018) propose that gender disparities in research productivity may stem from differences in access to advisor support, availability of assistantships, family responsibilities, and career aspirations. Lindahl et al. (2021) suggest that the size of collaborative networks is also a potential explanation. Xu and Shen (2025) further document a significant decline in the expected research output for married women with children, which is termed the “motherhood penalty.” They also find that higher socio-economic status provides a substantial buffer against the factors that contribute to gender gaps in research productivity. In addition to the gender gap, Roksa et al. (2022) find that students from underrepresented racial/ethnic groups tend to have lower publication rates in academic research, underscoring the importance of strengthening institutional support systems. Lindahl (2023) focuses on personality traits and finds that PhD students’ conscientiousness is positively related to research productivity and that this relationship remains similar across research areas.

Given the crucial role of supervisors in training PhD students (Mavrogalou-Foti et al., 2024), prior studies have also focused on how supervisors influence student research productivity (Lee, 2008; Li and Fernandez, 2025). Hilmer and Hilmer (2007) investigate the research productivity of PhD students in agricultural and resource economics programs. They find that those supervised by productive supervisors have more publications than their peers and that this effect is more pronounced for students who do their PhD studies at lower-ranked programs. García-Suaza et al. (2020) present similar findings and ascribe the effect to selection and differences in mentoring styles. When examining supervisor experience in mentoring, contrary to intuition, some studies report that supervisors’ mentoring experience is negatively associated with student research productivity, suggesting that supervisors may be more engaged or supportive in the early stages of their supervisory role (Corsini et al., 2022). Considering the intensive interaction needed for the transfer of complex knowledge and skills, student productivity may largely depend on their interactions with supervisors. Li and Fernandez (2025) explore the relationship between supervisor-student interactions and student productivity and find that, while whether students communicate with their advisers in person or remotely does not affect publication outcomes, supervisor availability, particularly communication frequency, is significantly and positively associated with higher research productivity.

This study uses contextual and institutional factors to describe the broader research environment, facilities, and institutions provided by universities or the academic community. One stream of this literature focuses on student peers, as peers play important roles in academic interactions, introducing peer pressure and stimulating knowledge exchange (Conti et al., 2014; Corsini et al., 2022). Some studies paid attention to supportive institutions. Horta et al. (2018) investigate the effects of funding on PhD student performance with a sample of doctorate holders residing in Portugal and find that those who received funding grants during their PhD studies are more productive than self-funded students. Pan et al. (2025) use a sample of PhD students in China and find that those who were selected for the CSC Program (i.e., an official program administered by the Chinese government to support PhD students in pursuing studies at overseas institutions) tend to be more productive than those who were not. Prior studies also underscore the importance of what the PhD program and the university can provide. Based on a sample of agricultural and resource economics PhD students, Hilmer and Hilmer (2007) document that those in top-ranked programs publish more articles, suggesting that academic resources and advisory quality are important in research activities. Belavy et al. (2020) find that PhD students in disciplines designated as strategic priorities by the university are likely to produce more publications. Overall, campus facilities and environment are important determinants of student outcomes (Bai et al., 2022).

With regard to interdisciplinary exposure, although some studies have demonstrated that engaging in interdisciplinary research fosters researchers’ productivity (Leahey et al., 2019; Leahey and Barringer, 2020; Chen and Wang, 2021), little is known about how interdisciplinary exposure is associated with PhD student research productivity. Millar (2013) finds that interdisciplinary dissertation research is associated with higher publication counts among US doctoral graduates. Pull et al. (2016) show that study field heterogeneity within research groups affects PhD completion rates. However, these studies do not directly address university-level disciplinary diversity or PhD student research productivity during doctoral training. As such, the question of how interdisciplinary exposure is associated with PhD student research productivity remains an open one.

### 2.2 Theoretical analysis

Academic research is a kind of activity aimed at expanding the boundaries of existing knowledge. In doing this, PhD students typically begin by acquiring foundational knowledge in a specific research field and then leverage university resources to explore cutting-edge topics and conduct original research. In the context of academic research, a key role of a university is to provide and coordinate the resources essential to research activities. This study borrows the resource-based theory from strategy management to construct a theoretical framework dealing with disciplinary diversity and student academic productivity. Strategy management scholars propose the resource-based view to justify why some organizations outperform others given the same external environment (Wernerfelt, 1984; Barney, 1991). They argue that the idiosyncratic resources and capabilities that constitute an organization are the basis of its competitive advantage, and that the management team maximizes their value by optimizing their allocation. An evolution of the resource-based view recognizes knowledge as a crucial input in production and considers the knowledge that an organization possesses as the most strategically important resource (Grant, 1996). Knowledge can be either explicit or tacit. Explicit knowledge is more informational and easier to acquire. Tacit knowledge, however, is experiential. It is developed through application and practice, making it difficult to imitate, substitute, or transfer (Grant, 1996; Ambrosini and Bowman, 2001). According to the view, managers of an organization should not only coordinate tangible resources but also establish systems to facilitate the acquisition, storage, transfer, and creation of knowledge. Drawing on this view, studies have made remarkable progress in understanding why some organizations are more successful in obtaining competitive advantage, especially in knowledge-related activities (Hayter, 2016; Chowdhury et al., 2022; Yang et al., 2025).

Academic research is a knowledge- and resource-intensive activity. From the resource-based view, to foster student academic activity, the university should try to provide favorable resources, such as advanced laboratories, powerful computing clusters, and digital subscriptions, among others. More importantly, it should establish supportive systems that enable effective acquisition, sharing, and transfer of knowledge from diverse sources. With the rapid development of science and technology, breakthroughs in academic research are more likely to emerge at the intersection of different disciplines (Wu et al., 2015; He et al., 2025). Prior studies point out that researchers are in need of interdisciplinary knowledge to extend the scope of their own research and enable innovation (Carayol and Thi, 2005; Wu et al., 2015; Pull et al., 2016; Vantard et al., 2023). He et al. (2025) use “academic cocoon” to describe the situation where a researcher’s information filtering mechanisms systematically ignore knowledge from other disciplines, resulting in repetitive research on topics similar to their prior work. They argue that being trapped in an academic cocoon hinders researchers from exploring complex questions and producing groundbreaking findings.

With respect to accounting research, interdisciplinary knowledge is also important. Accounting plays a pivotal role in organizations and society, thereby underscoring the need to understand it within its social and organizational context (Hopwood, 1983), and interdisciplinary research in accounting has been an important stream of accounting research (Jeacle and Carter, 2014; Dellaportas et al., 2022; Xu et al., 2023). It is argued that interdisciplinary knowledge and perspectives enrich accounting research by generating new ideas and flourishing research agendas (Jeacle and Carter, 2014; Xu et al., 2023). A typical interdisciplinary accounting research article generally relies on theories, hypotheses, or methods drawn from non-accounting disciplines (Hoffmann and Brivot, 2023). More specifically, Xu et al. (2023) conduct an analysis of 1,613 accounting research articles and report that these articles cite references from 131 disciplinary categories. As such, in a university with diverse disciplines, students would have more opportunities to be exposed to interdisciplinary knowledge and engage in collaboration with those from different disciplinary backgrounds, thereby combining their accounting knowledge with perspectives, theories, and methods from other disciplines to address novel accounting questions. Given the desirable role of interdisciplinary knowledge in accounting research, a positive relationship between the disciplinary diversity of universities and student research productivity is expected.

While interdisciplinary knowledge is crucial for academic research, resources are also indispensable. The total resources of a university are limited. If a university expands into too many disciplines, the resources each discipline receives would inevitably be reduced. This kind of resource-dilution effect is evidenced by some anecdotes. For example, in 2019, the University of Tulsa announced a restructuring plan that slashed its humanities programs to reallocate resources toward STEM and business disciplines due to financial constraints. Considering this resource-dilution effect, excessive disciplinary diversification of a university may cause a shortage of research resources available to students in each discipline, thereby reducing their academic productivity.

Taken together, this study predicts an inverted U-shaped relationship between university disciplinary diversity and PhD student research productivity. Put differently, this study posits that there is an optimal level of disciplinary diversity for a university. Below this level, student research productivity increases with disciplinary diversity; beyond it, productivity declines.

## 3 Research design

### 3.1 Sample selection

The data used in this study come from three sources. The first source is a database of PhD dissertations in accounting. Specifically, this study collects information on PhD students and their academic experience from their doctoral dissertations publicized on CNKI, one of the largest databases of digitized journal papers and dissertations in China. This study searches on CNKI for doctoral dissertations with the discipline major labeled as “Accounting” and finds 1,842 items in total. A typical dissertation contains useful information about, for example, the year of graduation, the duration of study, and the rank of the supervisor. Most importantly, as an established practice, PhD candidates generally include a section titled “Publications during the PhD program” or the like at the end of their dissertations to disclose their publications during the PhD studies. After excluding those without the disclosure of publications during the studies, this study ended up with 1,418 dissertations. The final sample comprises dissertations submitted by PhD candidates to 41 universities (or institutes), with graduation years spanning the period between 1999 and 2022.

The second source is the InCites database developed by Clarivate, which is a research analytics platform based primarily on the citation data and metadata from the Web of Science Core Collection. This study uses the publication data from InCites to measure the disciplinary diversity of universities. The third source is the public internet (e.g., university official websites), from which publicly available information about universities is collected.

### 3.2 Measuring student research productivity

Publication-based measures for research productivity are widely used in prior studies (Lindahl et al., 2021; Corsini et al., 2022; Lindahl, 2023; Li and Fernandez, 2025). This study thus measures student research productivity based on the publications disclosed in PhD dissertations. As mentioned, in the section “Publications during the PhD program”, students list their publications during their studies. Those publications typically contain journal papers, conference papers, and book chapters. This study focuses only on journal papers, as they generally undergo rigorous peer review. These journal papers can be in either English or Chinese. To ensure a basic level of academic standard of the publications, this study refers to two journal lists to screen these papers. One is the AGJ (Academic Journal Guide) list released by the Chartered Association of Business Schools to provide ratings for a wide range of English journals that publish papers from general business schools. The other is the CSSCI (Chinese Social Sciences Citation Index) list compiled by Nanjing University, which includes Chinese journals generally considered of high quality. The CSSCI list contains three series: the core collection, extended edition, and anthologized journals. This study takes all of them into account. The number of papers that have been published or accepted for publication in journals indexed in these two lists is used as a proxy for the research productivity of PhD students, labelled *ResearchProd*.

### 3.3 Measuring university disciplinary diversity

A plausible sign of a university’s disciplinary diversity is its active presence in research across a wide range of distinct disciplines. Therefore, this study leverages the number of research areas in which a university publishes papers to measure its disciplinary diversity. The InCites database captures the title, authors, publication year, journal name, author affiliations, and research area, among other metadata, for every paper it indexes. As for research area classification, it provides more than 20 predefined schemes, including the Web of Science subject categories and the China SCADC Subject 97 Narrow classification. The former comprises 254 research areas, while the latter, based on the subject categories established by China’s State Council Academic Degrees Committee, covers 97 research areas. This study primarily relies on the China SCADC Subject 97 Narrow classification to determine the research area of a paper.

Specifically, this study adopts the following procedures to calculate the university-level disciplinary diversity measure. (1) Downloading the publication records of each university between 1996 and 2022 from InCites. (2) Counting the number of papers published by a university in a specific research area over a rolling four-year window. This is because PhD students take an average of 4 years to complete their studies, and using rolling four-year data can better capture the disciplinary diversity they experienced during their time at the university. (3) This study focuses only on active research areas and discards those that account for less than 1% of the publications of a university. Excluding research areas in which a university rarely publishes helps mitigate noise stemming from sporadic publications. It is worth noting that using this threshold does not threaten the validity of the measure by excluding emerging fields. An emerging field that accounts for less than 1% of a university’s total output is, by definition, still marginal in terms of research activity. Its contribution to student interdisciplinary exposure is likely minimal. Meanwhile, this study uses a rolling four-year window. If an emerging field grows steadily, it will eventually exceed the 1% threshold and be counted as active. (4) Computing the proportion of active research areas out of a total of 97 research areas for each university and year as the proxy for disciplinary diversity, denoted *DisciplineDiv*.

A potential concern with this measure is that a paper’s journal subject category may not always match the administrative department of its author. For example, a professor appointed in management might publish in a computer science journal. This study treats such cross-disciplinary publications as valid signals of a university’s disciplinary diversity rather than measurement errors. The goal is to capture the diversity of knowledge to which students are exposed, not the formal administrative structure. While perfect alignment between journal categories and administrative units is not achieved, the measure remains an acceptable proxy for a university’s disciplinary portfolio.

### 3.4 Identification strategy

To identify the effect of university disciplinary diversity on student research productivity, this study uses Poisson regression to estimate the following equation:

$$\begin{array}{l} \text{ResearchPro}d_{i,t}=c+\text{αDisciplineDi}v_{i,t}+\text{βDisciplineDi}v_{i,t}^{2}\\ +\text{γControl}s_{i,t}+\delta_{t}+\varepsilon_{i,t} \end{array}$$ (1)

In Equation 1, *ResearchProd*_*i*,*t*_ is the dependent variable, representing the research productivity of student *i* who graduates in year *t*. *DisciplineDiv*_*i*,*t*_ is the disciplinary diversity of the university from which student *i* graduates in year *t*. *Controls*_*i*,*t*_ stands for a battery of control variables that may have an influence on student research performance. Specifically, the control variables are grouped into three categories. The first category captures student-specific information, including gender (labeled *Gender*, takes the value of 1 for male and 0 for female, inferred from the student’s name) and duration of study (*Duration*), defined as the number of years between enrollment and graduation.

The second category pertains to the student’s supervisor and includes two variables: *SupervisorRank*, which equals 1 if the supervisor is a full professor and 0 otherwise; *SupervisorTitle*, which takes the value of 1 if the supervisor was selected for the National Accounting Leadership Talent Development Program and 0 otherwise. This program is a prestigious national initiative aimed at cultivating top-tier accounting scholars. These two supervisor-specific variables are used to control for the potential influence of supervisors on student research productivity.

The third category contains variables related to the university. *FinancialSchool* is a dummy variable that equals 1 if the university is a specialized institution in finance and economics and 0 otherwise. *KeyDiscipline* is coded 1 if accounting (or its broader discipline, business administration) of the university is designated as a national key discipline, and 0 otherwise. These two variables serve as proxies for the institutional support and resource allocation that the university may provide to the accounting discipline. *DoubleFirstClass* is a dummy variable that takes the value of 1 if the university is included in the Double First-Class Initiative, and 0 otherwise. The Double First-Class Initiative is a major commitment made by the Chinese government to improve the international competitiveness of the universities. Including this variable helps capture the effect of a university’s overall resources on student research productivity. Considering that student research productivity may vary with time, this study also includes *δ_t_* to control for time fixed effects.

Since an inverted U-shaped relationship between university disciplinary diversity and student research productivity is expected, the coefficient of *DisciplineDiv*2_i,t_ is expected to be negatively significant.

### 3.5 Descriptive statistics

The descriptive statistics for the main variables are presented in Table 1. According to the statistics, the mean value of *ResearchProd* suggests that an accounting PhD candidate publishes 1.804 papers on average. The mean value of *DisciplineDiv* is 0.262, indicating that, on average, universities in the sample offer 26.2% of the disciplines specified by China’s State Council Academic Degrees Committee. Meanwhile, the disciplinary diversity of universities has been steadily increasing over the sample period. *Gender* has a mean of 0.549, indicating that 54.9% of the PhD students are male. The statistics for *Duration* show that a PhD student normally takes 4.087 years to complete the PhD program, while the shortest time is only 2 years and the longest time recorded is 12 years. Turning to the characteristics of supervisors, the results show that 98.2% of them are full professors, and 9% have been selected for the National Accounting Leadership Talent Development Program. As for the attributes of universities, the results indicate that 51.7% of them are specialized institutions in finance and economics, 40.2% of them have accounting designated as the national key discipline, and 72.4% are Double First-Class universities. Note that these attributes are not mutually exclusive; a single university may possess multiple characteristics. All the variables fall within a reasonable range.

**Table 1:** Descriptive statistics for main variables.

Variable	*N*	Mean	SD	Min	P25	Median	P75	Max
*ResearchProd*	1,418	1.804	2.114	0.000	0.000	1.000	3.000	14.000
*DisciplineDiv*	1,418	0.262	0.133	0.000	0.216	0.299	0.361	0.485
*Gender*	1,418	0.549	0.498	0.000	0.000	1.000	1.000	1.000
*Duration*	1,418	4.087	1.280	2.000	3.000	4.000	4.000	12.000
*SupervisorRank*	1,418	0.982	0.132	0.000	1.000	1.000	1.000	1.000
*SupervisorTitle*	1,418	0.090	0.286	0.000	0.000	0.000	0.000	1.000
*FinancialSchool*	1,418	0.517	0.500	0.000	0.000	1.000	1.000	1.000
*KeyDiscipline*	1,418	0.402	0.490	0.000	0.000	0.000	1.000	1.000
*DoubleFirstClass*	1,418	0.724	0.447	0.000	0.000	1.000	1.000	1.000

## 4 Results

### 4.1 Regression results of research productivity on disciplinary diversity

Table 2 presents the regression results. This study begins by fitting a simple linear regression to test for a monotonic relationship between university disciplinary diversity and student research productivity. In column (1), where the squared term of *DisciplineDiv* is not included, the coefficient of *DisciplineDiv* is statistically negative, suggesting that disciplinary diversity shows an aggregate negative effect on student research productivity. This implies a general pattern that when a university expands its disciplinary scope, the academic activity of a given discipline would be negatively influenced. In column (2), the squared term of *DisciplineDiv* is included to explore whether there is a non-linear effect between disciplinary diversity and student research productivity. The results show that the coefficient of *DisciplineDiv*^2^ is significantly negative, while the coefficient of *DisciplineDiv* is positive. This indicates an inverted U-shaped relationship between disciplinary diversity and student research productivity. Bases on the regression results, the inflection point of the inverted U-shaped relationship can be calculated as 0.194. This corresponds to approximately 19 active disciplines out of the 97 SCADC narrow subject categories. A plausible interpretation is that student research productivity increases with disciplinary diversity when *DisciplineDiv* is below 0.194 and decreases when it exceeds this value. This coincides with the resource-dilution effects. Moderate disciplinary diversity is associated with greater opportunities for interdisciplinary exposure, which may in turn contribute to student research productivity. However, once diversification exceeds an optimal threshold, resources allocated to a given discipline would be reduced, presumably leading to a decline in student research productivity.

**Table 2:** Regression results of research productivity on disciplinary diversity

Variables	*ResearchProd*	
	(1)	(2)
*DisciplineDiv*	–0.611*	4.036***
	(–1.938)	(3.574)
*DisciplineDiv* ^2^		–10.380***
		(–4.014)
*Gender*	0.159***	0.163***
	(2.812)	(2.862)
*Duration*	–0.045**	–0.050**
	(–2.062)	(–2.360)
*SupervisorRank*	0.271	0.228
	(1.038)	(0.850)
*SupervisorTitle*	0.340***	0.322***
	(4.179)	(4.060)
*FinancialSchool*	–0.159*	–0.137
	(–1.804)	(–1.599)
*KeyDiscipline*	–0.598***	–0.620***
	(–6.997)	(–7.636)
*DoubleFirstClass*	0.233**	0.149
	(2.347)	(1.552)
_cons	Included	Included
Year fixed effects	Included	Included
*N*	1,418	1,418
Wald chi^2^	807.921	843.920
Pseudo *R*^2^	0.136	0.142

Robust *z*-statistics are reported in parentheses. ***, **, and * represent significance at the 1%, 5%, and 10% levels.

The coefficients of control variables provide noteworthy insights. Regarding individual factors, male students tend to have more publications than their female counterparts at the end of their PhD studies, consistent with prior studies (Hilmer and Hilmer, 2007; Lindahl et al., 2021; Xu and Shen, 2025). Students who take a longer period of time to complete the PhD study are more likely to have fewer publications. One possible explanation is that challenges in degree completion may correlate with publication difficulties, potentially reflecting broader systemic or individual barriers. Regarding supervisory characteristics, the results reveal that students advised by supervisors with prestigious academic titles generally publish more journal papers. This may be attributed to supervisors’ established research networks and greater resource access. At the university level, the results show that students at universities specializing in finance and economics or designated as having accounting as a national key discipline publish fewer papers on average. This phenomenon may correlate with their advantageous job market competitiveness as compared to students from other universities who need to compensate through increased research outputs. Moreover, students from the Double First-Class universities tend to produce more publications, indicating the supportive effect of overall resources in research activities.

### 4.2 Effects of different types of disciplinary diversity

Variety, which my diversity measure primarily captures, is just one dimension of diversity, and balance and disparity are also important attributes of diversity (Stirling, 2007; Zhang et al., 2016; Yu et al., 2024). The baseline regression results demonstrate a negative effect of excessive disciplinary diversity on student research productivity, and this effect is hypothesized to be related to the diluted resources that a given discipline can receive. This study then performs analyses to verify this conjecture by reestimating the effects across subsamples with different types of disciplinary diversity.

#### 4.2.1 Effects of balanced versus imbalanced disciplinary diversity

Following Stirling (2007) and Zhang et al. (2016), this study uses the Gini coefficient to measure the balance of disciplinary diversity. In information science, the Gini coefficient is a well-known measure of concentration or evenness. For university *i* in year *t*, the balance of disciplinary diversity (Gini coefficient) is calculated with Equation 2:

$$\mathrm{Gin}i_{i,t}=1-\sum_{k=1}^{n}p_{k}^{2}$$ (2)

In the equation, *p_k_* represents the proportion of papers in discipline *k* among all papers published by the university. Similar to the calculation of disciplinary diversity, the Gini coefficient is also calculated over a rolling four-year window. A higher value of the Gini coefficient indicates more balanced disciplinary development. The sample is then split at the median of the Gini coefficient, creating a more balanced group and a less balanced group. This study then runs regressions to investigate the effect of disciplinary diversity on student research productivity in the two groups. Column (1) of Table 3 presents the regression results of the less balanced subsample. The coefficient of *DisciplineDiv* is statistically negative. By contrast, in column (2), where disciplines are more balanced, the coefficient of *DisciplineDiv* is positive. These results indicate that the negative effect of disciplinary diversity on student research productivity is concentrated in universities with a less balanced type of disciplinary diversity. This is consistent with the conjecture that imbalanced allocation of resources hampers research efficiency. The implication is that, when seeking to diversify its disciplinary portfolio, a university must guard against the continued concentration of resources in a few dominant disciplines.

**Table 3:** Effects of balanced versus imbalanced disciplinary diversity.

Variables	*ResearchProd*	
	(1)	(2)
	Disciplines less balanced	Disciplines more balanced
*DisciplineDiv*	–2.039***	0.335
	(–3.431)	(0.701)
*Gender*	0.198***	0.097
	(2.674)	(1.172)
*Duration*	–0.044*	–0.109***
	(–1.770)	(–2.678)
*SupervisorRank*	0.066	0.399
	(0.168)	(1.206)
*SupervisorTitle*	0.360***	–0.099
	(3.550)	(–0.579)
*FinancialSchool*	–0.105	–0.008
	(–0.964)	(–0.031)
*KeyDiscipline*	–0.273***	–1.490***
	(–2.632)	(–6.753)
*DoubleFirstClass*	0.216**	0.674**
	(2.178)	(2.338)
_cons	Included	Included
Year fixed effects	Included	Included
*N*	710	708
Wald chi^2^	437.014	607.925
Pseudo *R*^2^	0.150	0.200

Robust *z*-statistics are reported in parentheses. ***, **, and * represent significance at the 1%, 5%, and 10% levels.

#### 4.2.2 Effects of relevant versus irrelevant disciplinary diversity

As an attribute of diversity, disparity refers to the extent to which the members of a group differ from one another (Stirling, 2007; Zhang et al., 2016). When a university seeks to diversify its disciplines, it can choose to begin by developing new disciplines that are either closely aligned with or substantially distinct from its current portfolio. If it increases disciplinary diversity by developing relevant disciplines, it can make full use of its existing capabilities. If it diversifies into disciplines that are significantly different from the existing ones, the huge demand for resources of the new disciplines may lead to severe resource shortages. In China’s subject classification, accounting is positioned within the broader field of management, and management is generally regarded as a broader discipline with strong conceptual ties to economics. Therefore, this study constructs a variable to measure the degree to which a university’s disciplinary portfolio is closely related to accounting (*Disparity*), which is the ratio of disciplines in management and economics in which the university publishes to all disciplines it publishes in. This variable is also calculated using a rolling four-year window. A higher value of this variable means a lower level of disciplinary disparity, suggesting that the university tends to concentrate on disciplines that are relevant to accounting.

The sample is then partitioned into two groups based on the median value of disciplinary disparity, and the effects of disciplinary diversity on student research productivity in each group are reestimated. The results are reported in Table 4. Column (1) focuses on universities with low disciplinary disparity (i.e., relevant disciplinary diversity). The coefficient of disciplinary diversity is statistically insignificant. However, in column (2), where universities exhibit higher levels of irrelevant disciplinary diversity, the coefficient of disciplinary diversity is significantly negative. This indicates that if a university expands into disciplines that are distant from the existing ones, the benefits to student research are limited. This may be attributed to the crowding-out effect on resources and the reduced relevance of interdisciplinary knowledge. The results imply that diversifying disciplines centered on the existing ones may be an advisable strategy for universities.

**Table 4:** Effects of relevant versus irrelevant disciplinary diversity.

Variables	*ResearchProd*	
	(1)	(2)
	Low disciplinary disparity	High disciplinary disparity
*DisciplineDiv*	–0.790	–1.600**
	(–1.365)	(–2.338)
*Gender*	0.169**	0.127*
	(2.032)	(1.700)
*Duration*	–0.058*	–0.050*
	(–1.787)	(–1.738)
*SupervisorRank*	–0.074	0.921**
	(–0.244)	(2.527)
*SupervisorTitle*	0.414***	0.219
	(3.890)	(1.558)
*FinancialSchool*	–0.258**	–0.825**
	(–1.987)	(–2.259)
*KeyDiscipline*	–0.556***	–1.928***
	(–5.768)	(–4.555)
*DoubleFirstClass*	0.047	0.601*
	(0.447)	(1.664)
_cons	Included	Included
Year fixed effects	Included	Included
*N*	718	700
Wald chi^2^	388.361	615.680
Pseudo *R*^2^	0.135	0.201

Robust *z*-statistics are reported in parentheses. ***, **, and * represent significance at the 1%, 5%, and 10% levels.

### 4.3 Robustness checks

#### 4.3.1 Adjustments to measures of student research productivity

To make sure that the findings of this study are robust to different measures of student research productivity, this study makes two adjustments to the original measure of productivity. Specifically, this study first uses a stricter criterion to screen papers and only considers those published in journals included in the AJG list or the CSSCI core lists. This results in an alternative measure of student research productivity, labelled *ResearchProd_narrow*. This study also employs a broad criterion to screen papers, that is, taking all journal papers published by the student into consideration. The generated measure is denoted *ResearchProd_broad*. This study then reestimates Equation (1) with these two dependent variables and presents the results in columns (1) and (2) of Table 5. According to the results, the inverted U-shaped relationship between university disciplinary diversity and student research productivity is also observed.

**Table 5:** Results of robustness checks

Variables	*ResearchProd_narrow*	*ResearchProd_broad*	*ResearchProd*	
	(1)	(2)	(3)	(4)
*DisciplineDiv*	5.631***	1.802**		
	(4.419)	(1.988)		
*DisciplineDiv* ^2^	–13.382***	–8.103***		
	(–4.752)	(–3.909)		
*DisciplineDiv_adj*			–1.990***	
			(–3.706)	
*DisciplineDiv_adj* ^2^			–10.963***	
			(–4.037)	
*DisciplineDiv_WoS*				4.029***
				(2.592)
*DisciplineDiv_WoS* ^2^				–16.945***
				(–3.258)
*Gender*	0.137**	0.109**	0.161***	0.160***
	(2.276)	(2.166)	(2.833)	(2.847)
*Duration*	–0.060***	–0.036*	–0.050**	–0.045**
	(–2.679)	(–1.767)	(–2.343)	(–2.018)
*SupervisorRank*	0.090	0.118	0.223	0.269
	(0.316)	(0.583)	(0.832)	(1.053)
*SupervisorTitle*	0.385***	0.214***	0.322***	0.376***
	(4.449)	(3.061)	(4.059)	(4.648)
*FinancialSchool*	–0.247***	0.179**	–0.134	–0.236**
	(–2.617)	(2.261)	(–1.567)	(–2.098)
*KeyDiscipline*	–0.510***	–0.649***	–0.627***	–0.626***
	(–6.041)	(–8.242)	(–7.721)	(–7.398)
*DoubleFirstClass*	0.101	0.266***	0.155	0.181*
	(0.995)	(2.913)	(1.615)	(1.774)
_cons	Included	Included	Included	Included
Year fixed effects	Included	Included	Included	Included
N	1,418	1,418	1,418	1,418
Wald chi^2^	776.331	1213.971	844.764	820.647
Pseudo *R*^2^	0.144	0.141	0.142	0.138

Robust *z*-statistics are reported in parentheses. ***, **, and * represent significance at the 1%, 5%, and 10% levels.

#### 4.3.2 Adjustments to measures of university disciplinary diversity

Next, this study moves on to test the robustness to alternative measures of disciplinary diversity. One may argue that using data from InCites may introduce bias into the diversity measure because of the temporal trend of China’s universities increasing their publications indexed in InCites. To address this concern, this study calculates a new disciplinary diversity measure, which is adjusted for the average disciplinary diversity of all universities in that year, denoted *DisciplineDiv_adj*. Moreover, to enhance the generalizability of the study, this study employs the Web of Science subject categories as a substitute for the China SCADC Subject 97 Narrow classification to calculate the disciplinary diversity of universities, with the same procedures specified in subsection 3.3. This measure is labelled *DisciplineDiv_WoS*. Using these two independent variables, this study reruns the regressions and reports the results in columns (3) and (4) of Table 5, which are qualitatively similar to the baseline regressions.

Collectively, these robustness checks consolidate the findings that university disciplinary diversity has an inverted U-shaped effect on student research productivity.

### 4.4 Effects of disciplinary diversity on other dimensions of student research performance

So far, this study has documented both the positive and negative effects of university disciplinary diversity on student research productivity. To advance our understanding of how disciplinary diversity influences student research performance, this study proceeds to examine the effects of disciplinary diversity on student research interdisciplinarity and research impact.

#### 4.4.1 Effect of disciplinary diversity on research interdisciplinarity

In the theory section, this study argues that diversified disciplines foster the exchange and integration of interdisciplinary perspectives, theories, and methods, which, if true, would increase the likelihood of students conducting interdisciplinary research. This study thus investigates whether disciplinary diversity increases the interdisciplinarity of doctoral dissertations. This study follows Hoffmann and Brivot (2023) to determine whether a student commits to interdisciplinary accounting research by reviewing the doctoral dissertation and checking if it relies on theories, hypotheses, or methods from disciplines outside accounting. The variable is denoted *InterdiscResearch*. This study employs a Logit model to examine the effect of disciplinary diversity on research interdisciplinarity and presents the results in column (1) of Table 6. The coefficient of disciplinary diversity turns out to be statistically positively significant. The results show that PhD students at universities with greater disciplinary diversity are more likely to choose interdisciplinary studies, which is consistent with the conjecture that interdisciplinary exposure is positively associated with students’ engagement in interdisciplinary research.

**Table 6:** Effects of disciplinary diversity on student research performance

Variables	*InterdiscResearch*	*ResearchImpact*
	(1)	(2)
*DisciplineDiv*	1.300*	0.004**
	(1.946)	(2.475)
*Gender*	0.063	0.000
	(0.516)	(0.514)
*Duration*	0.066	–0.000
	(1.342)	(–1.281)
*SupervisorRank*	0.117	0.001*
	(0.233)	(1.930)
*SupervisorTitle*	0.169	–0.001**
	(0.781)	(–2.165)
*FinancialSchool*	–0.321	0.001
	(–1.542)	(1.601)
*KeyDiscipline*	0.022	–0.000
	(0.134)	(–0.590)
*DoubleFirstClass*	–0.185	0.002***
	(–0.928)	(3.665)
_cons	Included	Included
Year fixed effects	Included	Included
*N*	1,418	1,418
Wald chi^2^/*F*	49.505	24.720
Pseudo/Adjusted *R*^2^	0.031	0.194

Robust *z-* or *t-*statistics are reported in parentheses. ***, **, and * represent significance at the 1%, 5%, and 10% levels.

#### 4.4.2 Effect of disciplinary diversity on research impact

The impact of interdisciplinary research is debated. On one hand, interdisciplinary research may achieve a greater impact as it can attract a broader audience and draw citations from scholars in more disciplines (Leahey et al., 2016; de Bakker et al., 2019). On the other hand, however, some interdisciplinary research may lack depth in any one discipline and thereby makes scholars reluctant to cite (Xu et al., 2023). To provide new evidence for this debate, this study performs analyses to explore how disciplinary diversity shapes the impact of doctoral dissertations. To enable comparability across dissertations published in different years, this study measures the impact (*ResearchImpact*) of a dissertation as its citations divided by downloads. This study employs an OLS method to estimate the effect of disciplinary diversity on research impact. The regression results presented in column (2) of Table 6 show that disciplinary diversity has a positive effect on research impact. This means that students empowered by interdisciplinary knowledge appear to produce more impactful research. Two possible reasons may explain this relationship: First, students trained in an environment of diversified disciplines are more likely to choose interdisciplinary research topics that naturally attract broader audiences and receive more citations. Second, as doctoral dissertations, they overcome the limitations of other publications that merely apply interdisciplinary knowledge superficially, thereby ensuring higher research quality.

### 4.5 Time-based heterogeneity analysis

To examine whether the main findings change with time, this study conducts a time-based heterogeneity analysis. 2017 is selected as the breakpoint because of the launch of the Double First-Class Initiative. As mentioned, this initiative substantially increased resources for selected universities. The sample is split into two cohorts: students who enrolled before 2017 and those who enrolled in 2017 or after. The Poisson regression is reestimated separately for each subsample. The results are reported in Table 7, indicating that the inverted U-shaped relationship remains similar across different time spans.

**Table 7:** Regression results for pre-2017 and post-2017 enrollment cohorts.

Variables	*ResearchProd*	
	(1)	(2)
	Before 2017	In and after 2017
*DisciplineDiv*	4.939***	6.559**
	(3.888)	(1.962)
*DisciplineDiv* ^2^	–13.026***	–12.692**
	(–4.388)	(–1.967)
*Gender*	0.141**	0.204*
	(2.195)	(1.807)
*Duration*	–0.060**	–0.100
	(–2.506)	(–0.777)
*SupervisorRank*	0.261	–0.041
	(0.808)	(–0.090)
*SupervisorTitle*	0.281***	0.511***
	(2.933)	(3.014)
*FinancialSchool*	–0.117	–0.094
	(–1.228)	(–0.417)
*KeyDiscipline*	–0.628***	–0.661***
	(–6.813)	(–3.455)
*DoubleFirstClass*	0.101	0.293*
	(0.888)	(1.647)
_cons	Included	Included
Year fixed effects	Included	Included
*N*	1,160	258
Wald chi^2^	643.094	268.862
Pseudo *R*^2^	0.136	0.226

Robust *z*-statistics are reported in parentheses. ***, **, and * represent significance at the 1%, 5%, and 10% levels.

Notably, the inflection point is 0.190 for the pre 2017 cohort and 0.258 for the post 2017 cohort. As a benchmark, the point calculated with the baseline estimate is 0.194. A plausible explanation is that the additional resources allocated under the Double First-Class Initiative relaxed the resource dilution constraint, allowing a slightly wider optimal disciplinary scope. This finding is consistent with the resource-based view. When resources increase, the trade-off between disciplinary diversity and resource dilution shifts in favor of diversity. Overall, these results confirm the temporal stability of the core findings and offer preliminary evidence that resource injections can shift the optimal diversity level upward.

## 5 Discussion

Against the backdrop of growing interdisciplinary integration, this study investigates whether exposure to diverse disciplines is associated with the research productivity of PhD students, an area that remains largely unexplored. Three specific research questions are investigated in this study, and the findings offer novel insights and carry meaningful implications for both academic research and doctoral education policy.

First, as for how university disciplinary diversity is associated with PhD student research productivity, the results reveal an inverted U-shaped relationship between university disciplinary diversity and student research productivity. Student research productivity peaks when a university is active in approximately 19 out of 97 disciplinary categories. Universities with a narrower disciplinary scope may benefit from careful expansion into neighboring fields, as the marginal benefit of interdisciplinary exposure likely outweighs the resource cost. In contrast, universities with a very broad scope should be aware that further diversification may dilute resources and reduce student output. Notably, the time-based heterogeneity analysis shows that the inverted U-shaped relationship holds both before and after the 2017 Double First-Class Initiative, with the optimal level of disciplinary diversity increased from 0.190 to 0.258. This suggests that additional resources relaxed the dilution constraint, shifting the balance toward greater diversity. Therefore, the optimal diversity level is not fixed but depends on the university’s resource availability. However, these numbers are derived from a sample of Chinese accounting PhD students and should be interpreted as illustrative rather than universal. Given that universities differ in their resource endowments, existing research strengths, and strategic priorities, each university should identify its own optimal level of disciplinary diversity based on these contextual factors. Meanwhile, considering that the mere presence of a diversified disciplinary portfolio does not guarantee that PhD students will experience meaningful interdisciplinary exposure, institutional facilitators, such as interdisciplinary courses, cross-departmental colloquiums, and co-advising frameworks, also play a critical role. Universities should therefore establish systems to foster meaningful interdisciplinary exposure.

Inspired by Stirling (2007), Zhang et al. (2016), and Yu et al. (2024), who include balance and disparity as important attributes of diversity, the second goal of the study is to establish how the balance and disparity of the diversified disciplines are associated with student research productivity. The results show that the negative effects of disciplinary diversity on student research productivity are observed only in the subsample of universities with greater disciplinary imbalance. This indicates that imbalanced allocation of resources hampers the effectiveness of interdisciplinary academic research. Therefore, when seeking to diversify discipline portfolios, universities should implement mechanisms to prevent resources from being monopolized by a few disciplines. Regarding the disparity attribute, the results indicate that the negative effects of disciplinary diversity on student research productivity are concentrated among universities that expand into too many irrelevant disciplines. This aligns with the intuition that knowledge from distant disciplines is difficult to integrate for new knowledge production, and that diversifying into irrelevant disciplines may crowd out resources allocated to existing ones. These results highlight the critical roles of both disciplinary balance and relevance in shaping the outcomes of disciplinary diversity and may help explain why some universities fail to achieve the expected gains from disciplinary diversity. Success of disciplinary diversification hinges on balanced resource allocation across fields and cognitive proximity between new and existing disciplines. Without these, expanding the disciplinary portfolio risks diluting student research productivity rather than enhancing it.

Finally, this study proceeds to look into the impact of university disciplinary diversity on two additional dimensions of student research performance, namely, the interdisciplinarity and impact of doctoral dissertations. An interdisciplinary doctoral dissertation is defined as one that employs theories, hypotheses, or methods from disciplines outside accounting (Hoffmann and Brivot, 2023). The results from a Logit model show that PhD students at universities with greater disciplinary diversity are more likely to choose interdisciplinary studies. This is consistent with the conjecture that exposure to interdisciplinary knowledge enables students to conduct interdisciplinary research. Moreover, the study finds that university disciplinary diversity has a positive effect on the citations of doctoral dissertations. This means that students benefiting from interdisciplinary knowledge tend to produce more impactful academic research. Taken together, these findings reveal the value of interdisciplinary knowledge to the research performance of PhD students and contribute to a comprehensive understanding of the effects of disciplinary diversity at the student level. These findings carry two practical implications. For universities, a diverse disciplinary climate can help students undertake interdisciplinary research. For doctoral students, actively seeking interdisciplinary exposure beyond their home departments could enhance both the novelty and the visibility of their work.

To summarize, this study sheds light on how interdisciplinary exposure shapes student research activities and offers practical implications for university strategic planning. However, it has some limitations. The first limitation is its generalizability. The findings are derived from accounting PhD students in Chinese universities. Accounting is a business and social science discipline. Its research processes, publication norms, and the nature of interdisciplinary knowledge integration may differ substantially from the natural sciences, engineering, or humanities. Therefore, readers should be cautious about extrapolating the specific magnitude of effect, inflection point or the exact shape of the relationship to other disciplines. Actually, accounting research already routinely incorporates theories, methods, and data from economics, management, psychology, and sociology. This means that accounting PhD students may already have a baseline level of interdisciplinary exposure even in a low diversity university. Consequently, the additional benefit of increasing university disciplinary diversity may be smaller in accounting than in a more siloed discipline. If this is true, the estimates of the positive slope and the inflection point may be conservative. Future research is encouraged to reexamine the relationship in other disciplines. Second, due to the lack of micro-level data on, for example, interdisciplinary coursework enrollment records or formal co-supervision from professors outside the home department, the results reported in this study is correlational. Future research with micro-level data is needed to establish causality. Another limitation concerns the measurement of research productivity. This study uses the number of journal papers listed in recognized journal indices as a proxy for productivity. While this measure captures publication quantity with a quality screen, it does not account for other important dimensions of research quality, such as author contribution and innovativeness of research. Future research should collect more granular data on authorship and research quality to extend these findings.

## 6 Conclusion

This study is among the first to investigate how a university’s disciplinary diversity influences the research productivity of PhD students. The findings are threefold. First, the relationship between university disciplinary diversity and student research productivity has an inverted U-shaped form, suggesting that a modest degree of disciplinary diversity is beneficial for new knowledge generation, but excessive diversification can be harmful. Second, the negative effects of disciplinary diversity on research productivity are concentrated on the types of diversification that ignore disciplinary balance and relevance. Third, disciplinary diversity is found to increase the likelihood of students choosing interdisciplinary research topics as their PhD studies and improve research impact.

Taken together, the findings suggest adopting a dialectical perspective on disciplinary diversity. Universities are encouraged to maintain an adequate level of disciplinary diversity, which fosters knowledge exchange and transfer but avoids resource dilution. In doing so, universities should ensure the balance of different disciplines and prioritize diversification into fields closely aligned with their existing strengths. For PhD students, the implication of this study is that being open to interdisciplinary knowledge helps identify research ideas and produce new knowledge.

## Data Availability

The raw data supporting the conclusions of this article will be made available by the authors, without undue reservation.
